# Supplementary material for: Adult mortality before and during the first wave of COVID-19 pandemic in nine communities of Yemen: a key informant study
Source: Confl Health. 2022 Dec 12;16:63. doi: 10.1186/s13031-022-00497-3 (PMC9743127; doi:10.1186/s13031-022-00497-3)

Adult mortality before and during the first wave of COVID-19 pandemic in nine communities of Yemen: a key informant study

# SUPPLEMENTARY FILE

# Record linkage criteria

Table S1. Record linkage criteria and corresponding match classifications.

| Key variables | | | Supporting variables | | | | Overall match decision |
| --- | --- | --- | --- | --- | --- | --- | --- |
| Difference in names | Difference in year of death (y) | Difference in age at death (y)† | Gender | Address at death | Cause of death | Locality of origin |  |
| If 2-4 names are available:  all are the same  OR  If 4 names are available:  3/4 are the same and the names are in the same order | <2 | <10 | same | same | same | same | definite |
|  |  | <10 | same | at least one variable is the same | | | probable |
|  |  | 10-14 | same | same | same | same | probable |
|  |  | 15-19 | same | same | same | same | possible |
|  | 2-4 | <15 | same | same | same | same | probable |
|  |  | 15-19 | same | same | same | same | possible |
|  | ≥5 | <20 | same | same | same | same | possible |
|  | any | any, but age ≥60yo at death | Variables not considered | | | | possible |
| If 4 names are available:  3/4 are the same but the names are in a different order  OR  If 3 names are available:  2/3 are the same and the names are in the same order | <5 | <10 | Variables not considered | | | | probable |
| If 3 names are available:  2/3 are the same but the names are in a different order | <2 | <5 | Variables not considered | | | | possible |

† If the age at death of either decedent being matched was <20yo, the cut-offs for tolerable differences were halved.

# Descriptive characteristics and list overlap by study site

Graphics titles in order:

1. Number of deaths reported by lists over the years.

2. Number of deaths reported by lists for each age group.

3. Percentage of female vs male decedents reported by lists.

4. Overlap among decedents lists.NB: Burial preparers (in the main text) and corpse washers (in the additional file) refer to the same category of key informants.

## Site A1


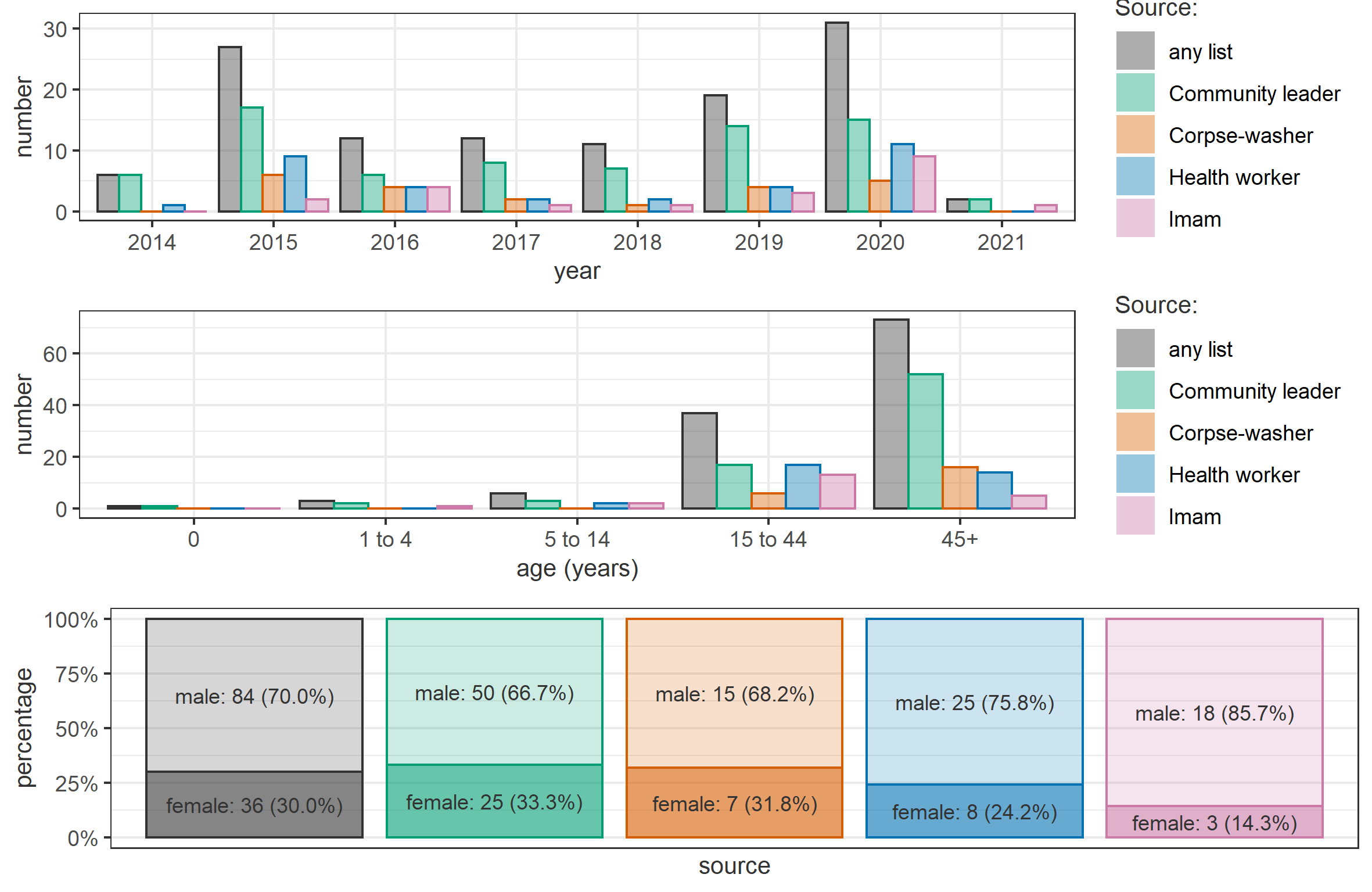


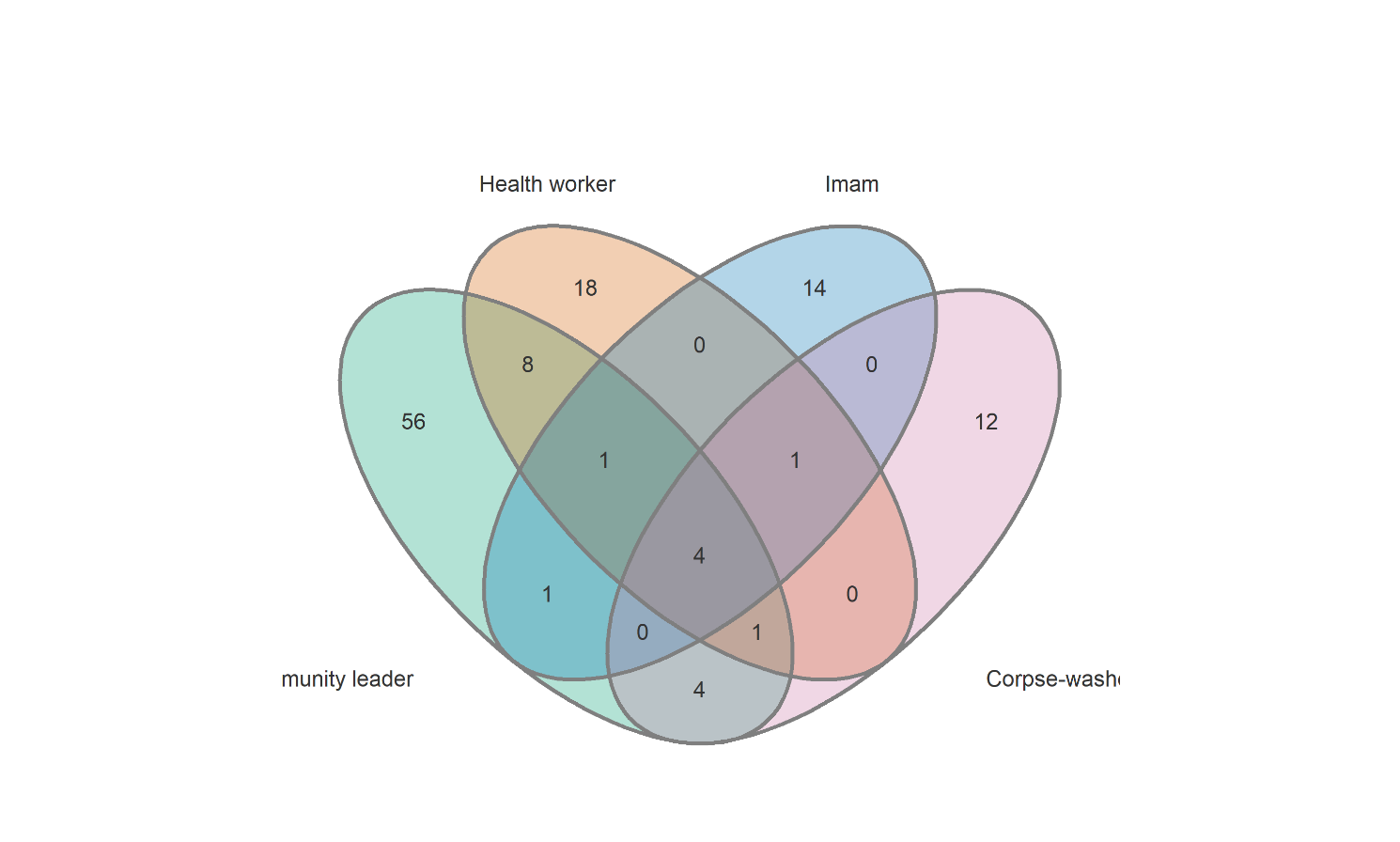


## Site A2


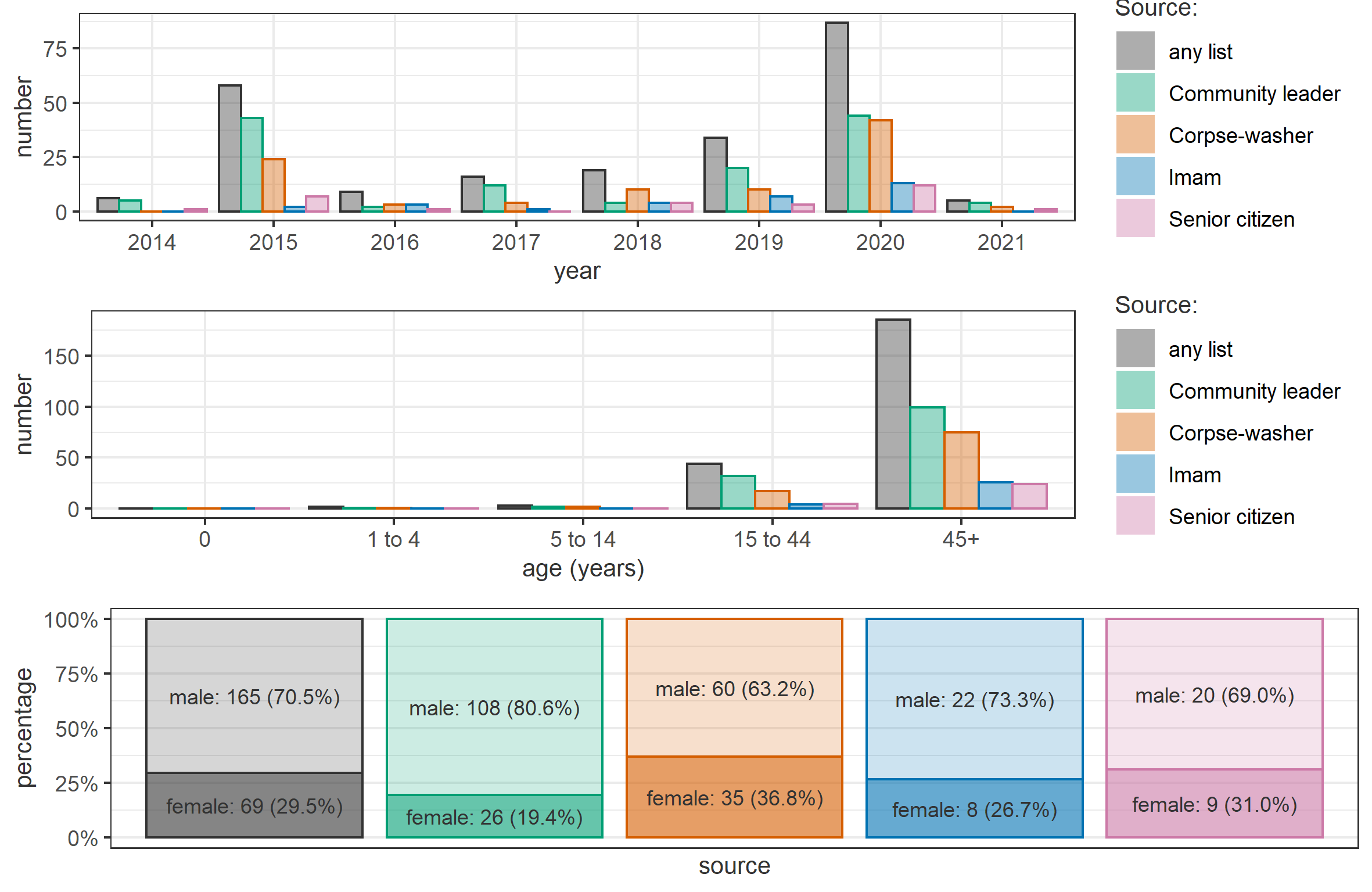


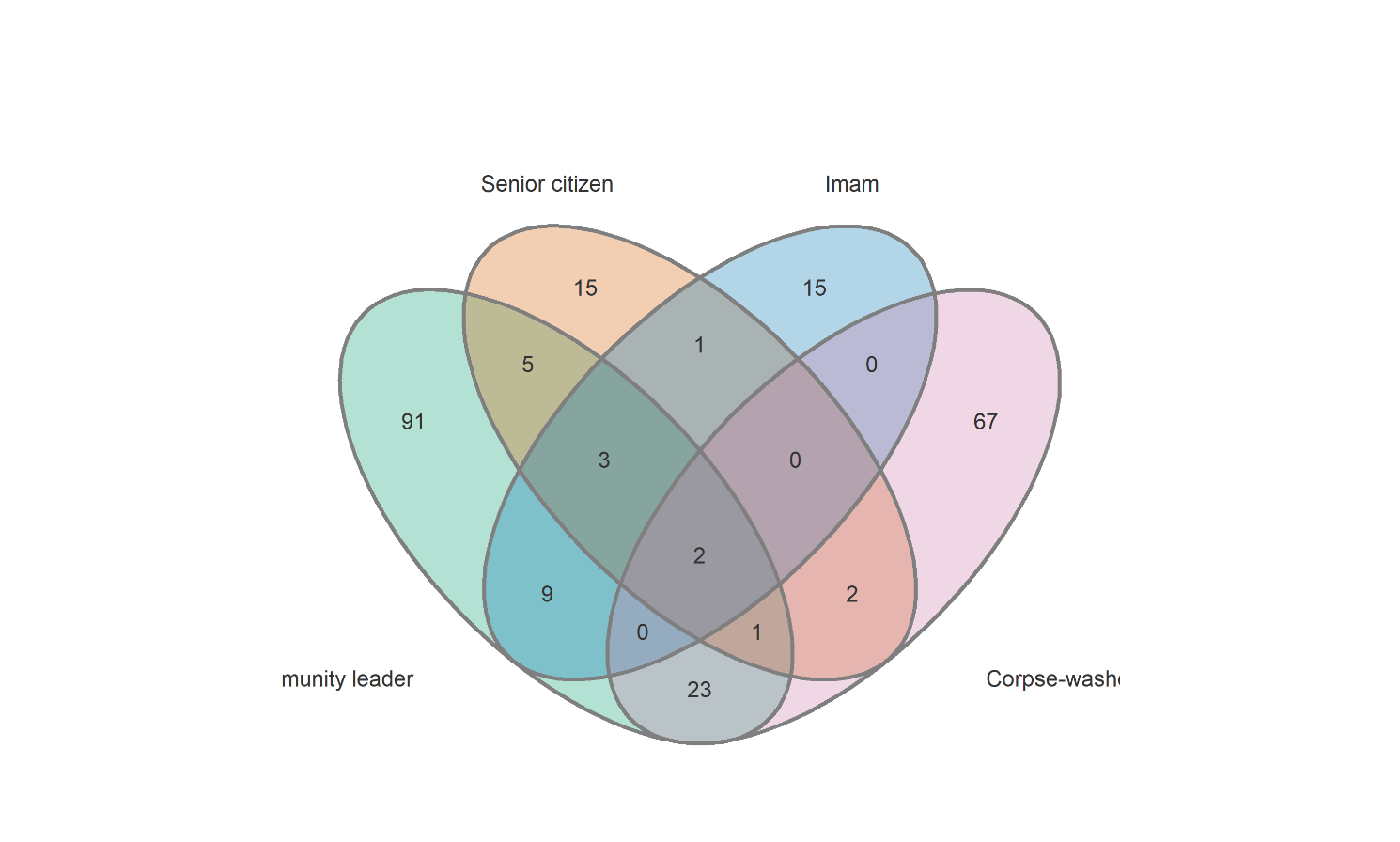


## Site A3


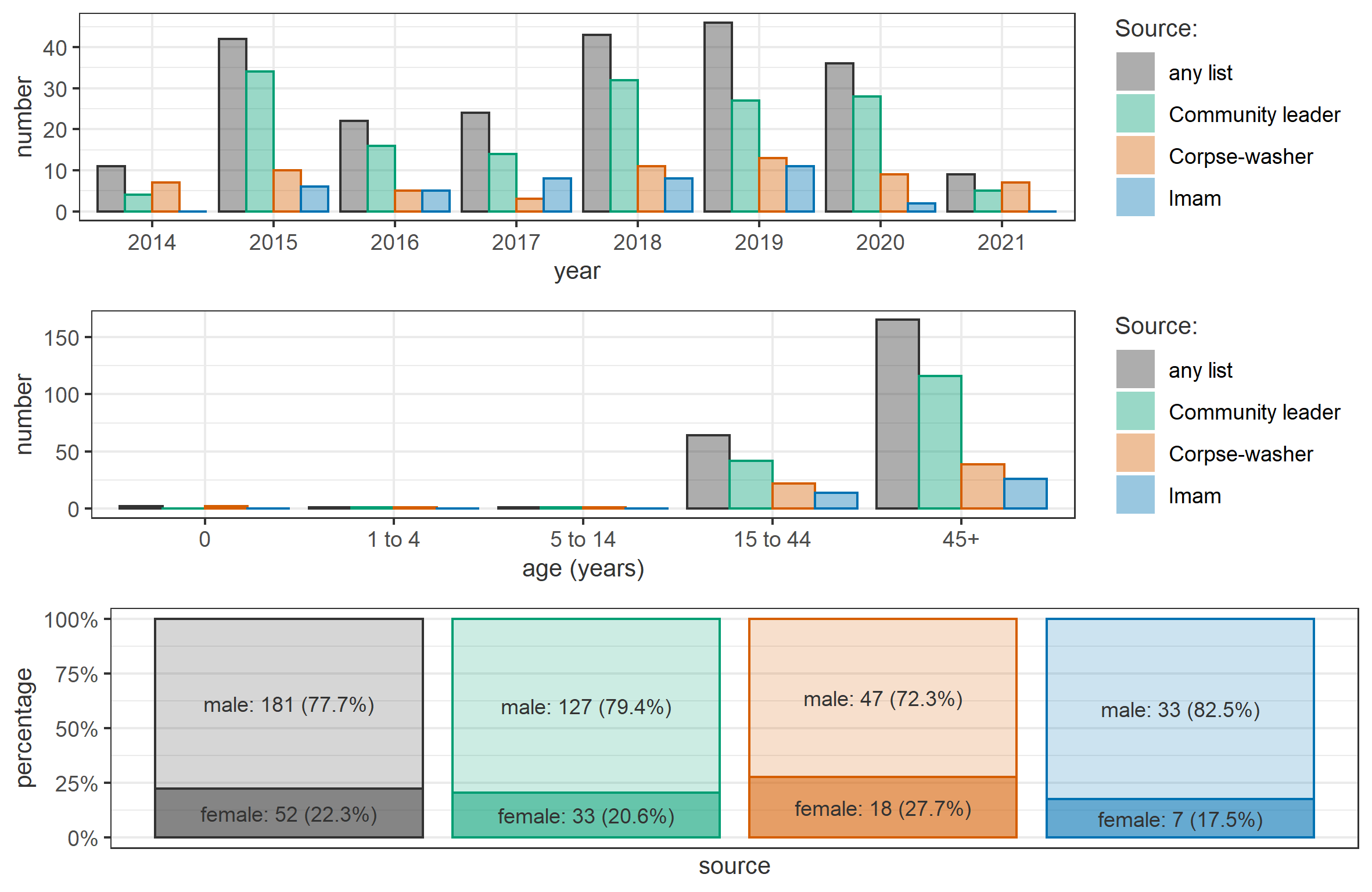


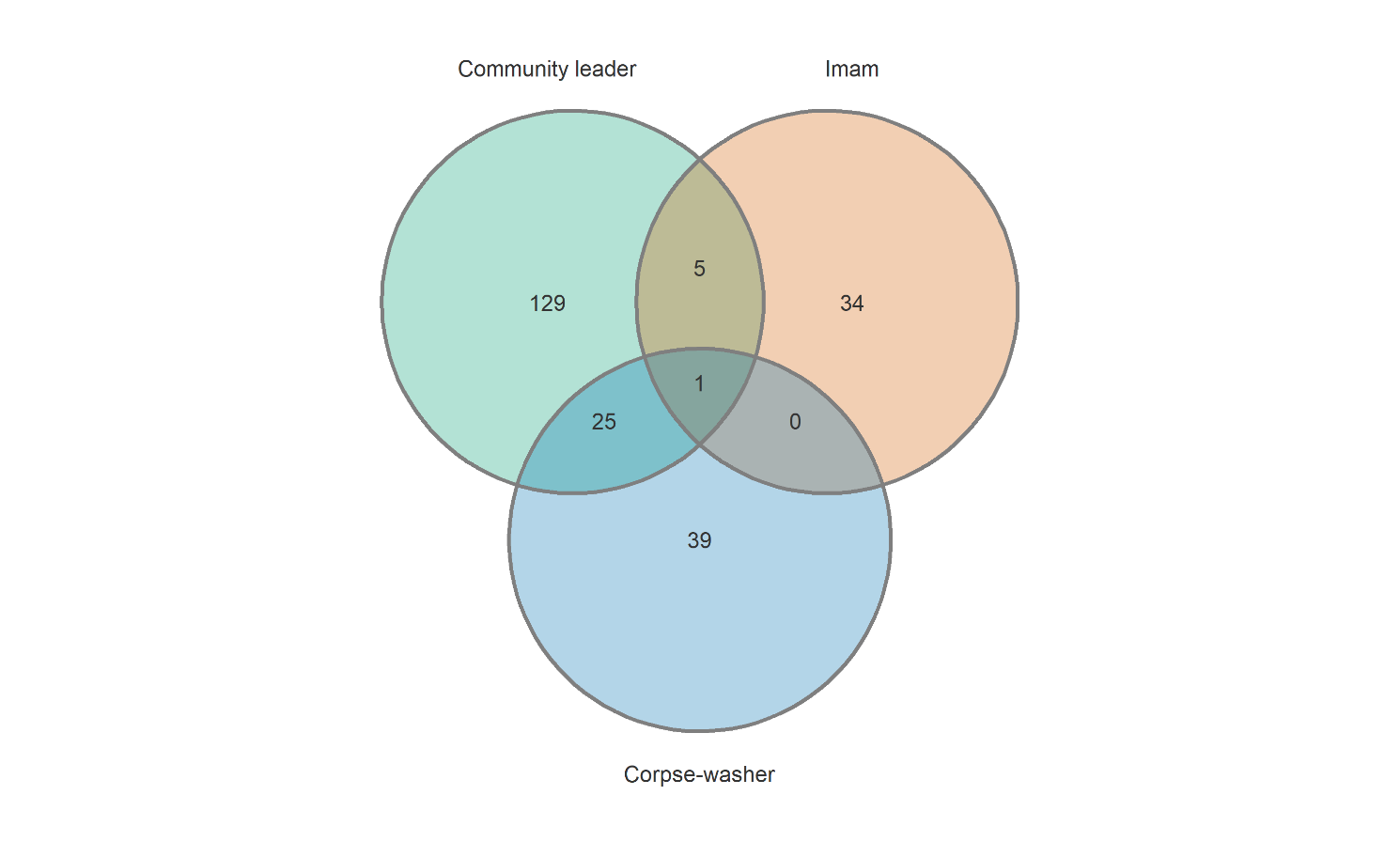


## Site A4


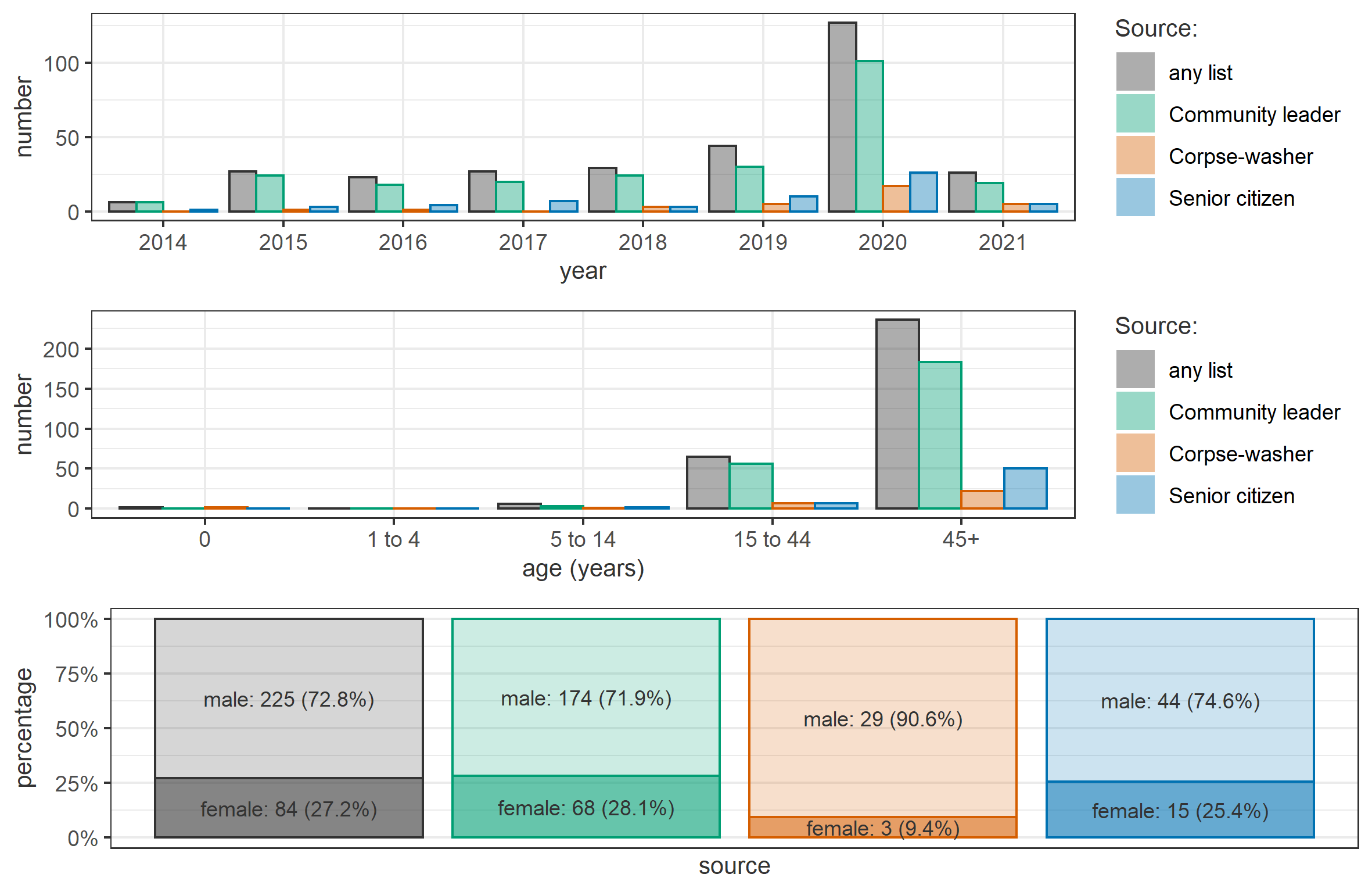


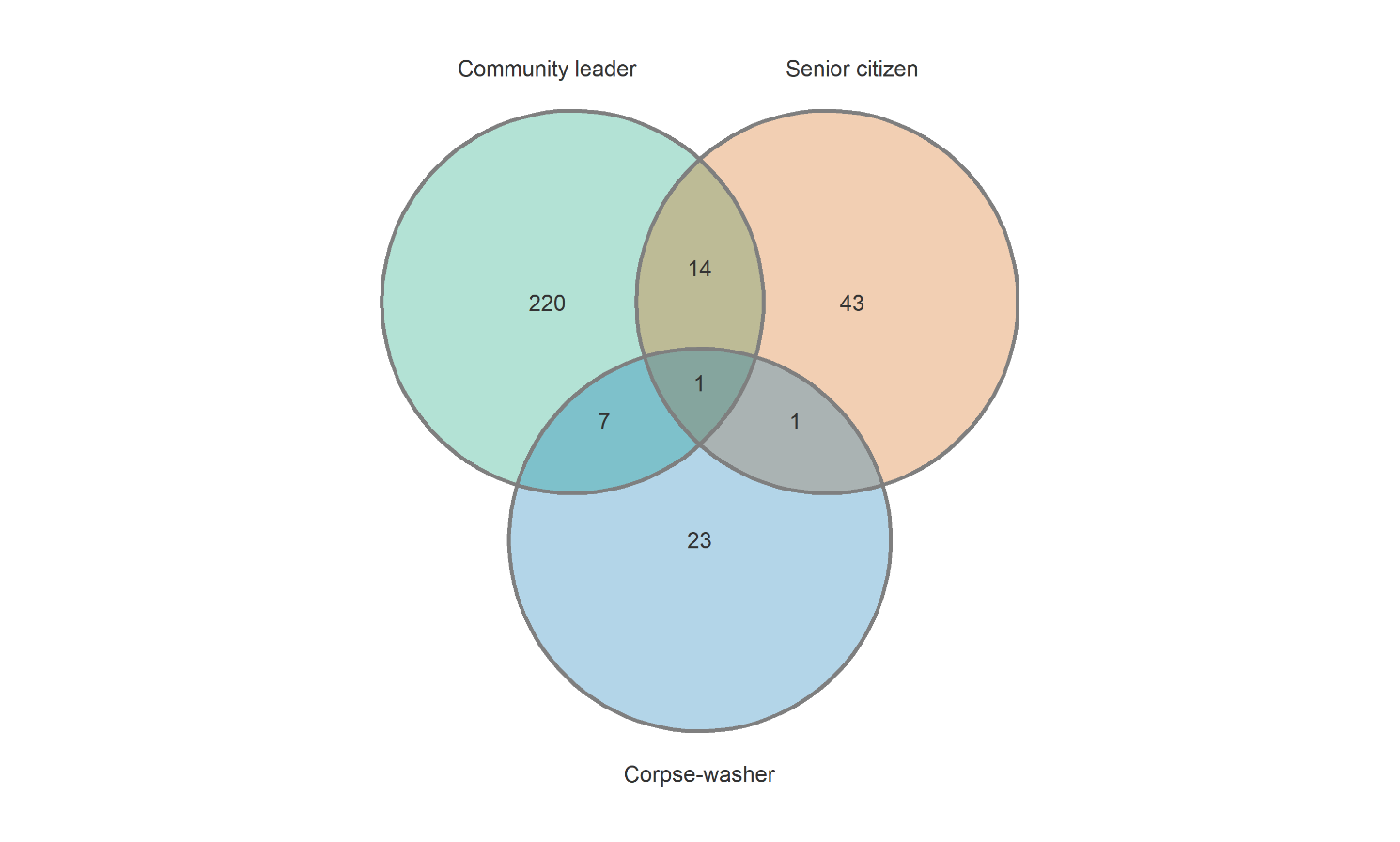


## Site T1


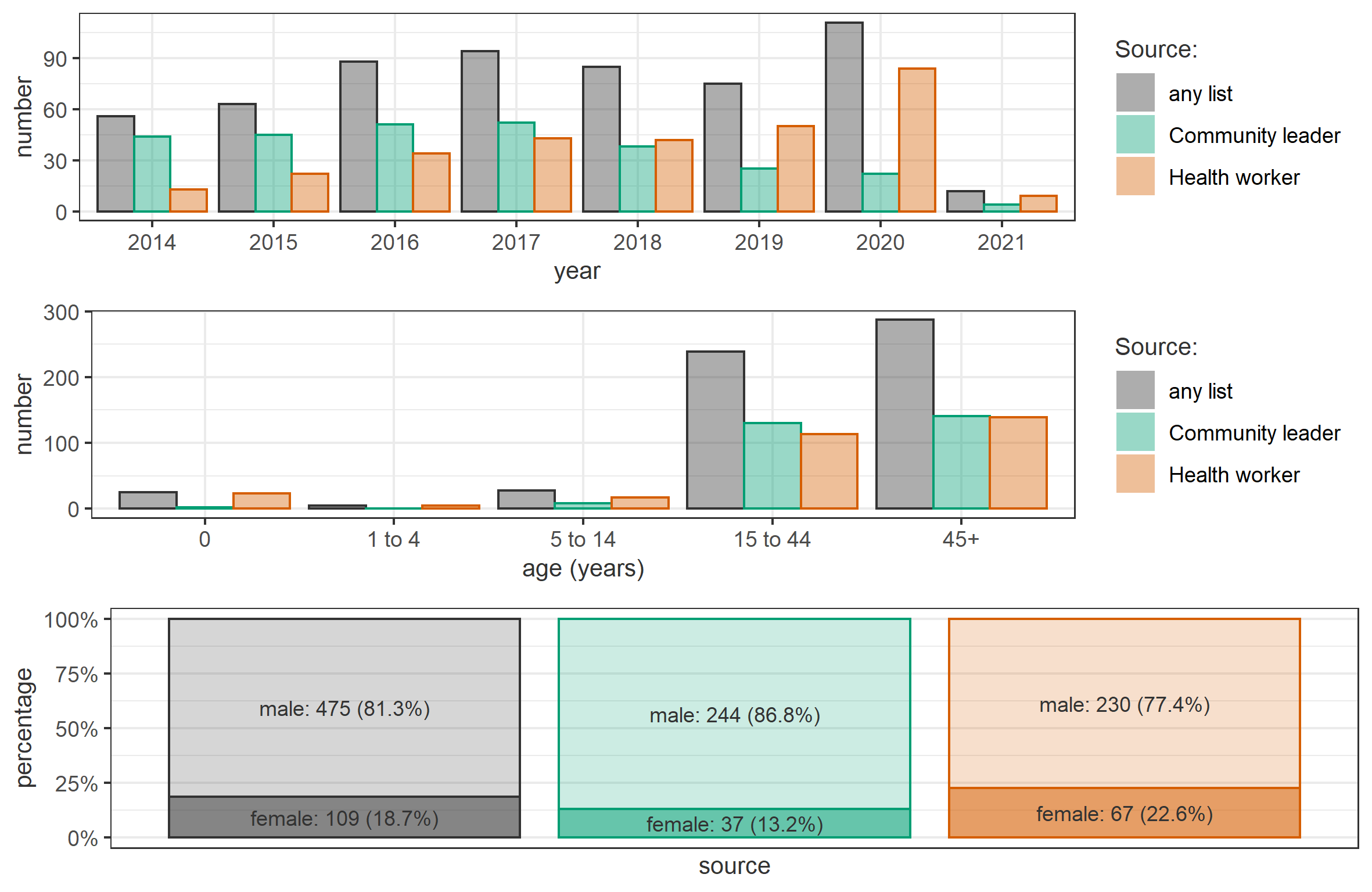


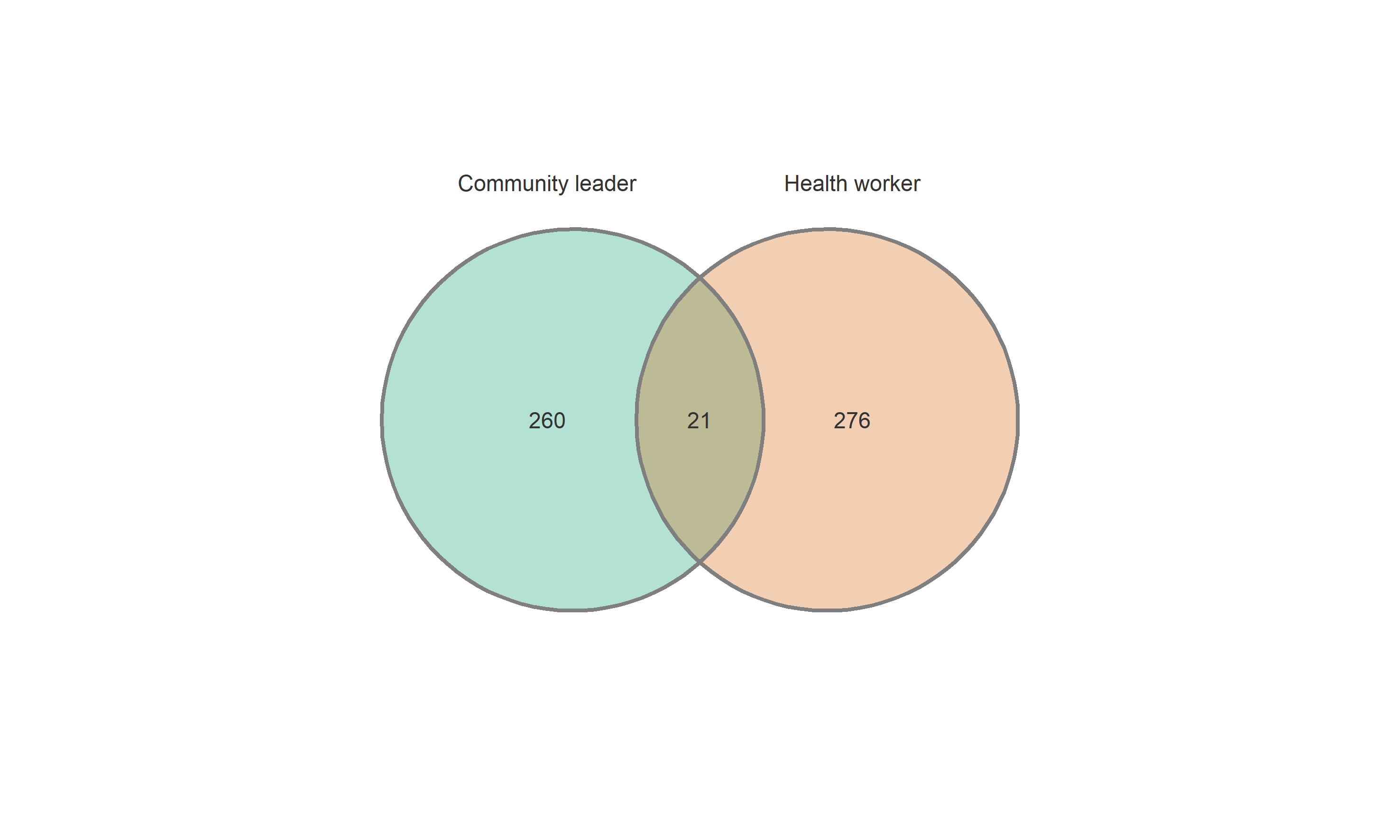


## Site T2


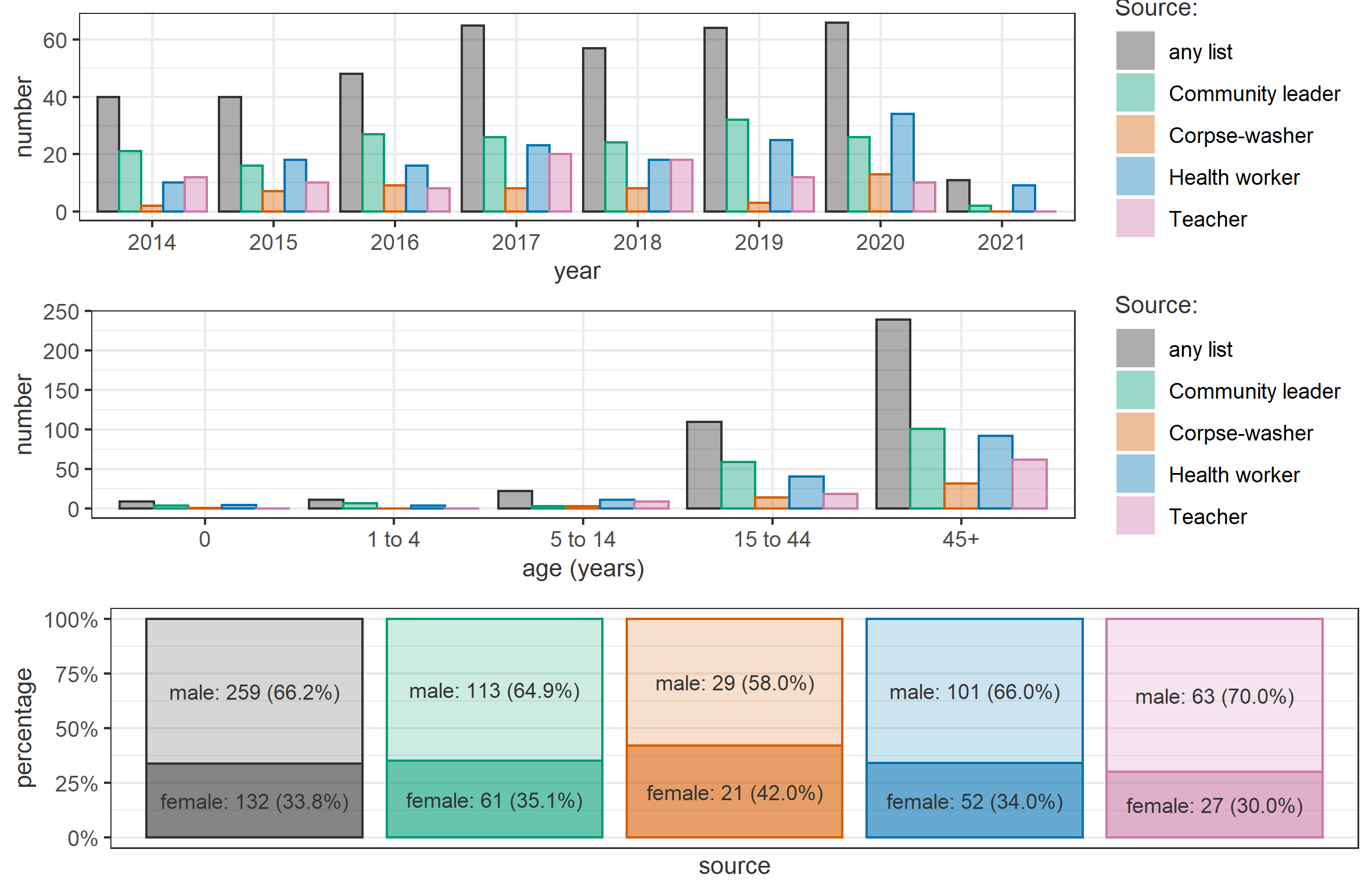


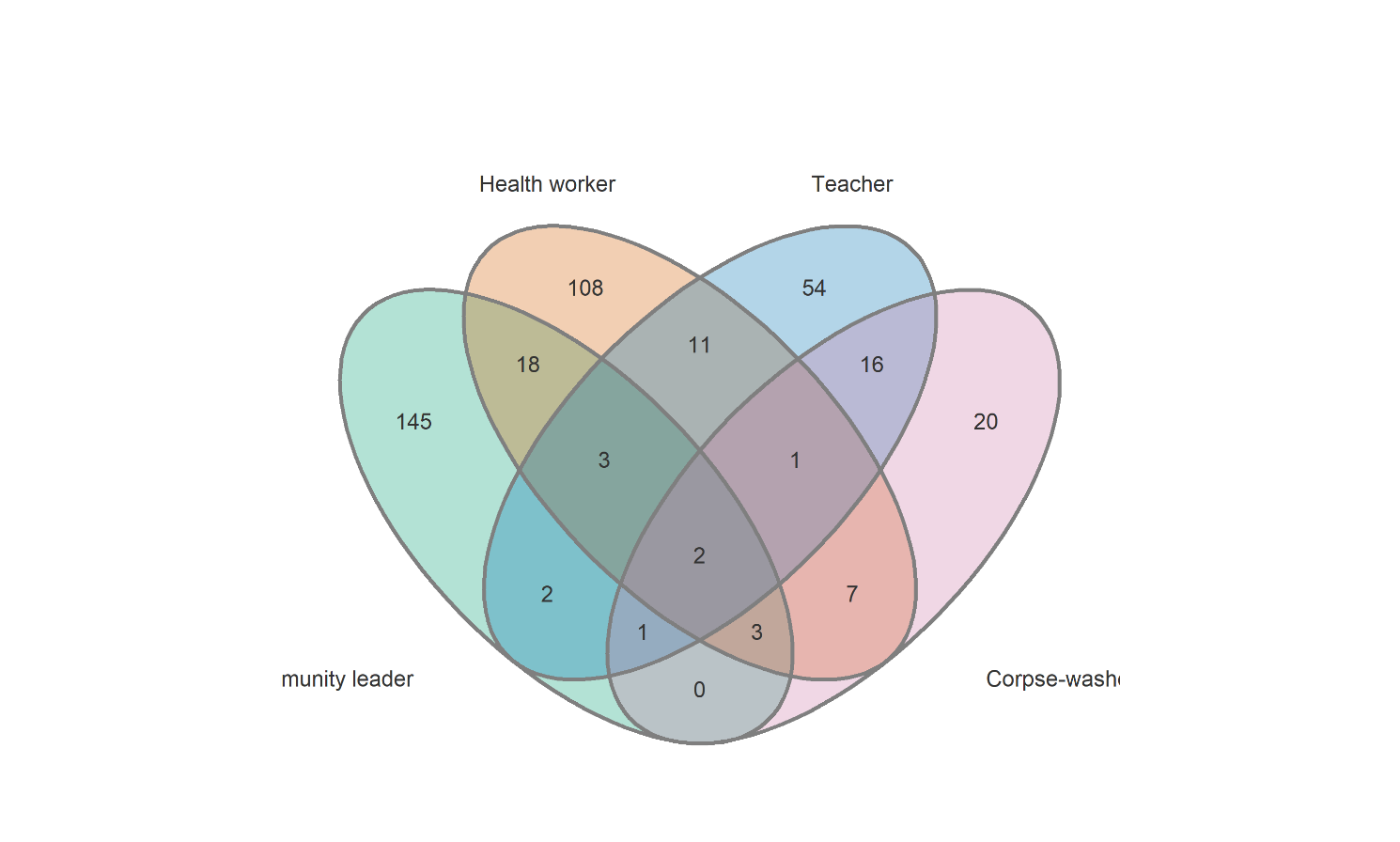


## Site T3


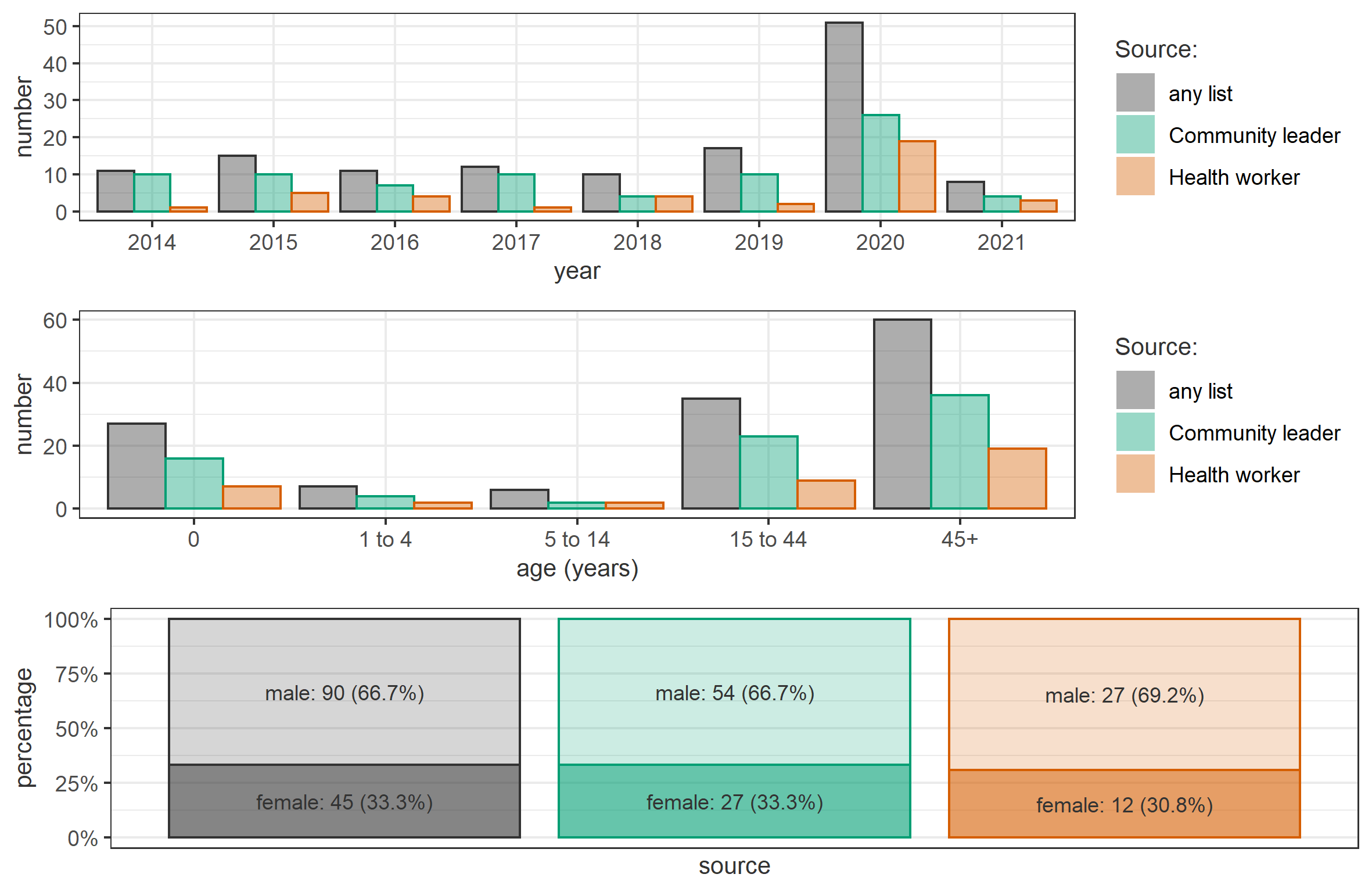


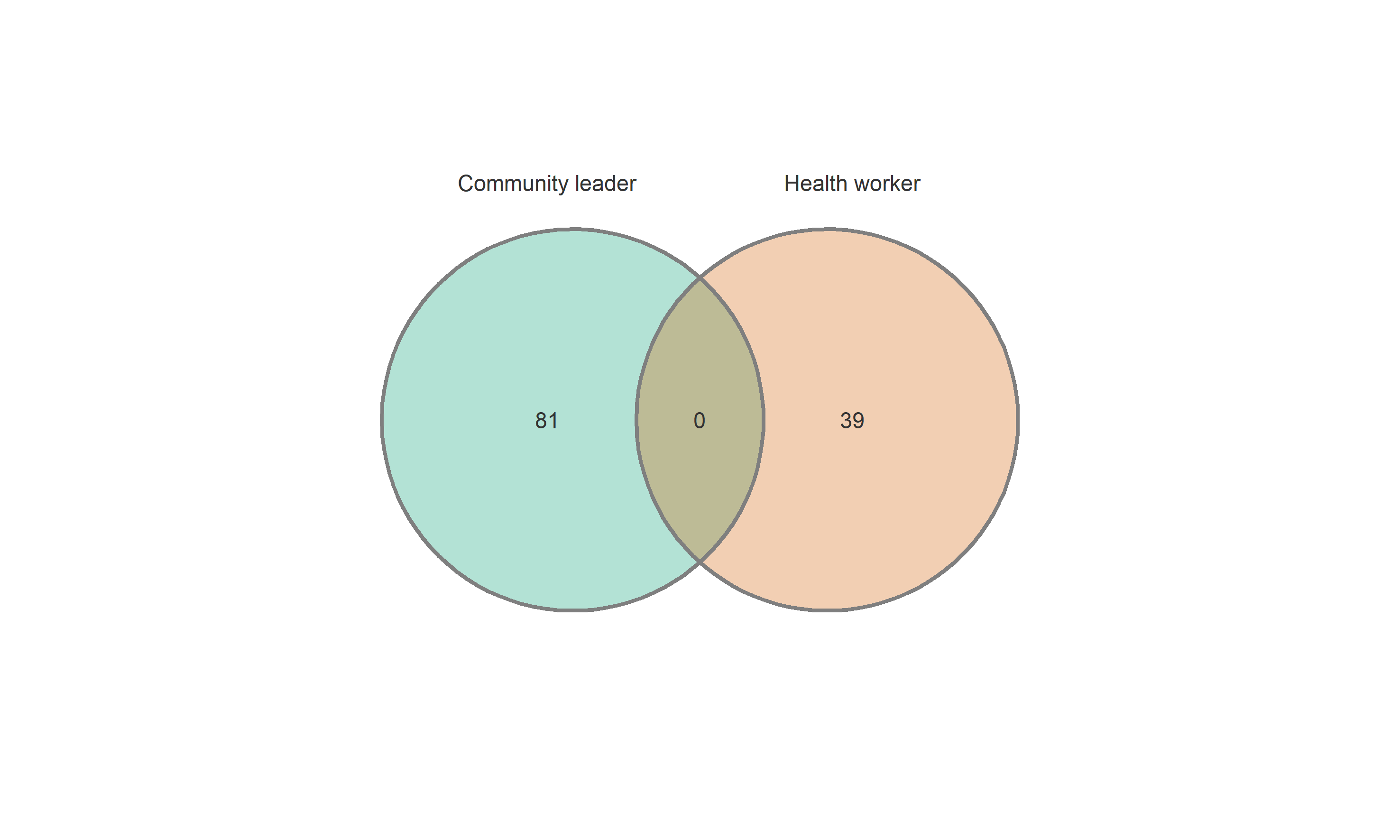


## Site T4


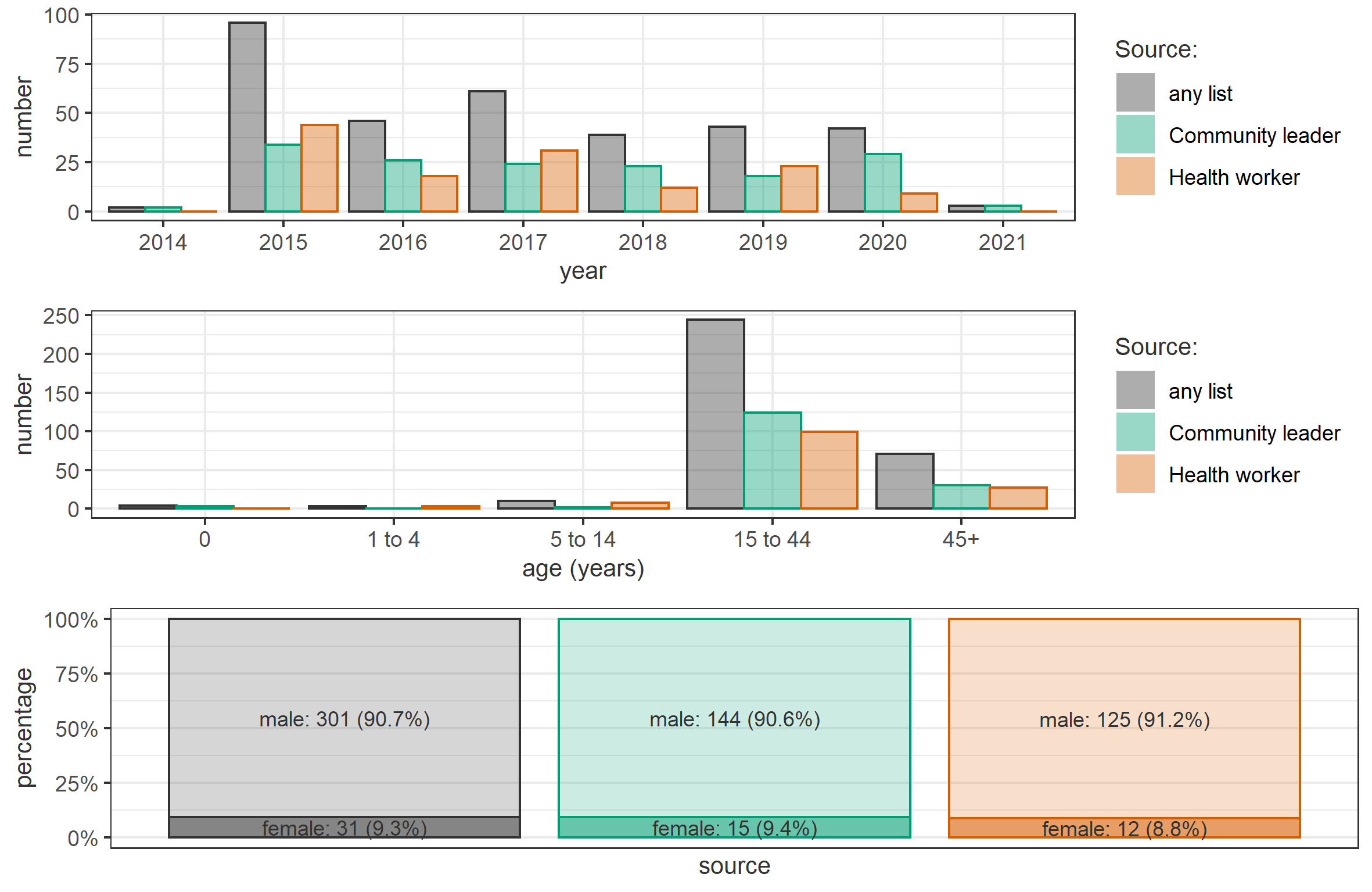


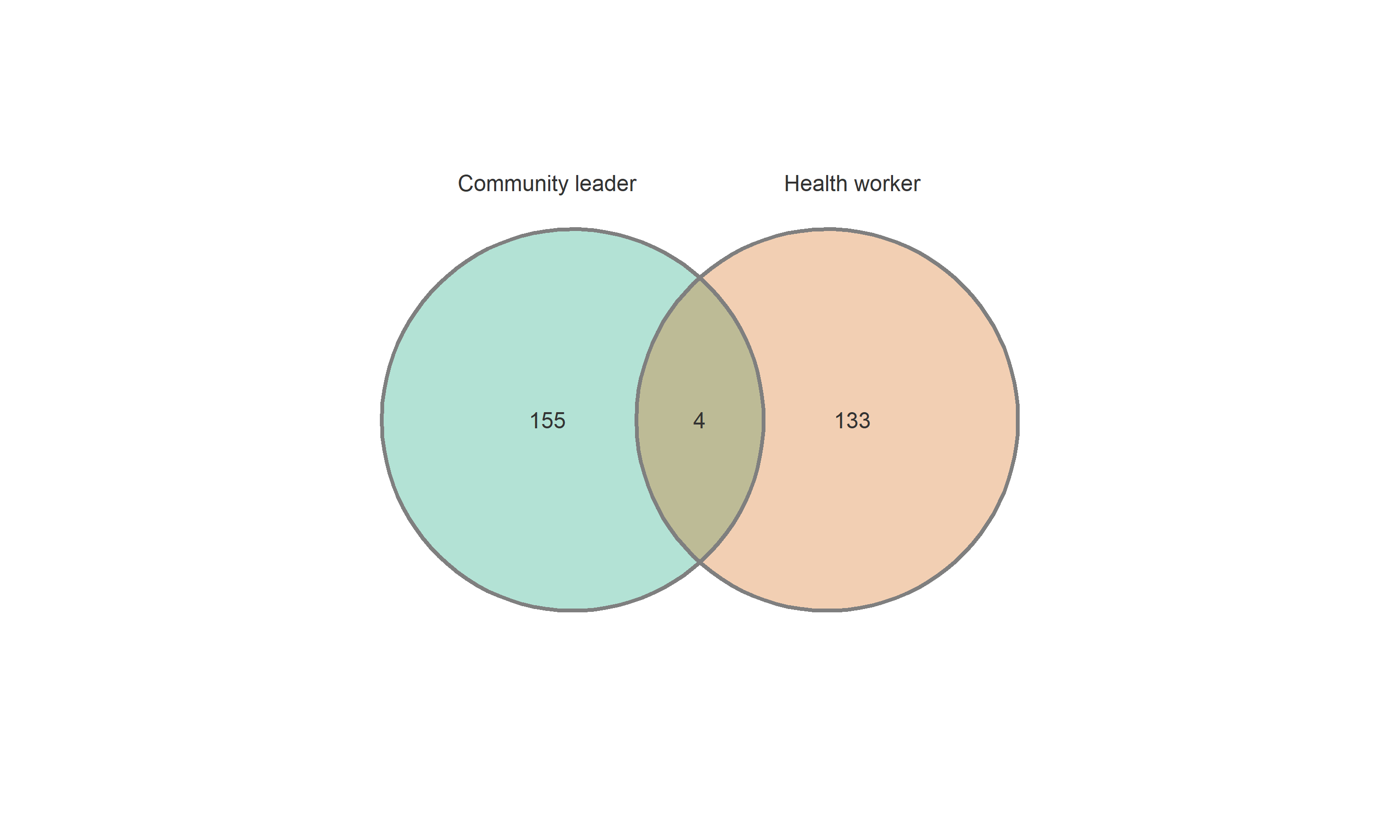


## Site T5


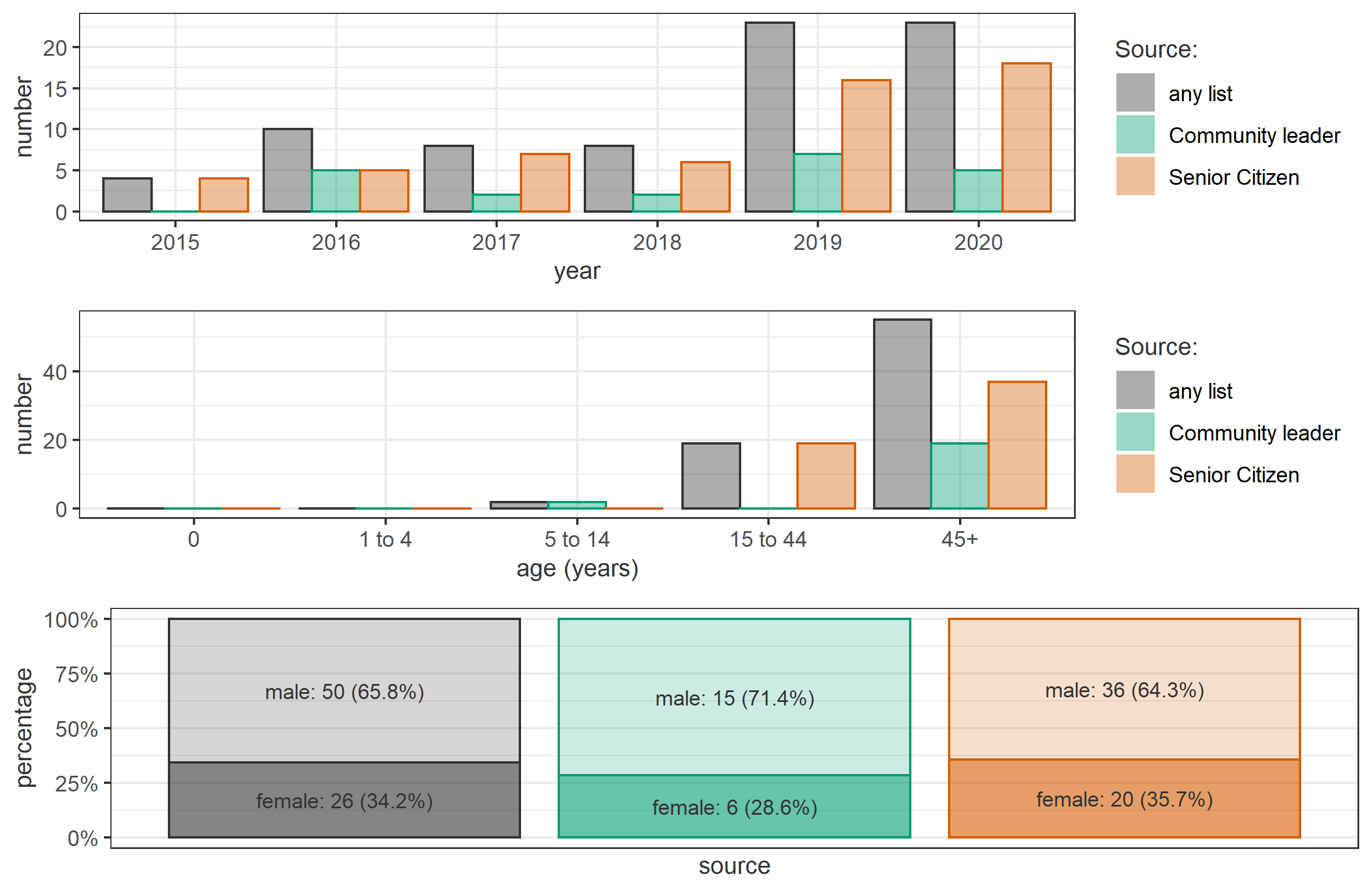


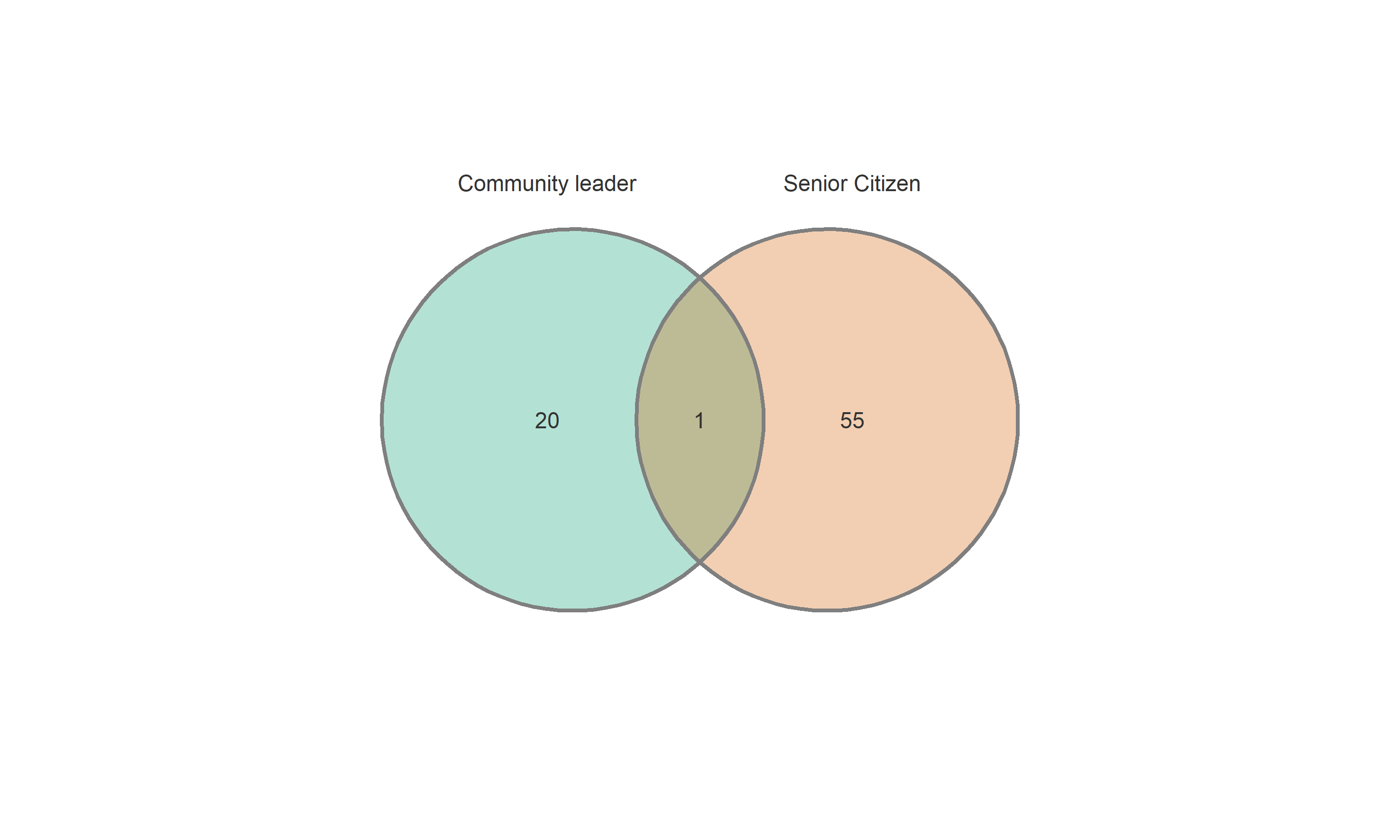

Supplement: Supplementary file 1 — Additional file 1. Record linkage criteria, descriptive characteristics and lists overlap by study site. [file 13031_2022_497_MOESM1_ESM.docx]
